# Safety of micronized palmitoylethanolamide (microPEA): lack of toxicity and genotoxic potential

**DOI:** 10.1002/fsn3.392

**Published:** 2016-06-15

**Authors:** Earle R. Nestmann

**Affiliations:** ^1^ Health Science Consultants Inc.MississaugaOntarioL5N 7S2Canada

**Keywords:** Acute, Ames test, micronized, micronucleus, mutagenicity, palmitoylethanolamide, subchronic toxicity

## Abstract

Palmitoylethanolamide (PEA) is a natural fatty acid amide found in a variety of foods, which was initially identified in egg yolk. MicroPEA of defined particle size (0.5–10 *μ*m) was evaluated for mutagenicity in *Salmonella typhimurium,* for clastogenicity/aneuploidy in cultured human lymphocytes, and for acute and subchronic rodent toxicity in the rat, following standard OECD test protocols, in accordance with Good Laboratory Practice (GLP). PEA did not induce mutations in the bacterial assay using strains TA1535, TA97a, TA98, TA100, and TA102, with or without metabolic activation, in either the plate incorporation or liquid preincubation methods. Similarly, PEA did not induce genotoxic effects in human cells treated for 3 or 24 h without metabolic activation, or for 3 h with metabolic activation. PEA was found to have an LD50 greater than the limit dose of 2000 mg/kg body weight (bw), using the OECD Acute Oral Up and Down Procedure. Doses for the 90‐day rat oral toxicity study were based on results from the preliminary 14‐day study, that is, 250, 500, and 1000 mg/kg bw/day. The No Effect Level (NOEL) in both subchronic studies was the highest dose tested.

## Introduction

The history of PEA as a natural food ingredient with medicinal properties has been described by Masek and Raskova (1967). The initial observation was in 1943 by Coburn et al. (1943), as part of an epidemiological study focused on childhood rheumatic fever, the incidence of which was higher in those children consuming diets low in eggs. These investigators noted that occurrence was reduced in children fed egg yolk powder, and subsequently they demonstrated antianaphylactic properties in guinea pig with a lipid extract from egg yolk (Coburn et al. 1954).

Subsequently, a crystalline anti‐inflammatory agent was isolated from egg yolk as well as from soybean lecithin (Kuehl et al. 1957). They identified the chemical structure to be N‐2 hydroxyethyl palmitamide, or PEA. Further studies on the anti‐inflammatory, antianaphylactic, antiserotonin, and antihistamine effects of PEA were conducted by Ganley et al. (1958) and Ganley and Robinson (1959). It was later found that PEA is an endogenous compound, locally synthesized in animal and human tissues and body fluids, to protect against perturbing inflammation (Skaper et al. 2013, 2014).

The purpose of this study is to review the safety of PEA, in addition to publishing new toxicology data, to establish a robust safety profile for the use of this substance in health products intended for human and veterinary consumption. Background information stems from the early recognition of PEA as a potential therapeutic agent. A wide‐ranging drug development program was initiated by the Institute of Pharmacology of the Czechoslovak Academy of Sciences, whose efforts are documented in a comprehensive, unpublished English summary by Professor Karel Masek, M.D., Dr. Sc., Head of the Institute (Masek 1980). These and many other pharmacological and clinical studies were conducted in support of the clinical development of PEA as a pharmaceutical agent named IMPULSIN^®^ (Spofa) and also marketed in Spain under the name Palmidrol (Laboratorie Prodes, S.A.). The studies have been summarized in three lengthy appendices: (1) background, safety studies, and pharmacology, (2) clinical studies, and (3) references and approval of manufacturing in 1970 by the Czech Ministry of Health (Masek 1980).

In light of the fact that these previous studies are not well documented or published, new GLP studies as described in this study have been performed to establish a more robust safety profile for PEA. The results of genetox assays as well as acute and repeat dose, oral toxicity studies in the rat will be described. The new studies have been performed using a PEA preparation of defined purity and particle size, on the basis of recent evidence suggesting that particle size is a relevant issue in defining biological consequences of PEA oral administration (Skaper et al. 2013; Impellizzeri et al. 2014).

## Materials and Methods

### Laboratory

GLP (Good Laboratory Practice) tests (Intox, 2015a,b,c,d,e, 2016) were carried out in the test facility of INTOX Pvt. Ltd. in Maharashtra, India (Intox), on behalf of study sponsor Prismic Pharmaceuticals of Scottsdale, Arizona (Prismic). Intox has been certified as a GLP compliant laboratory both by the National GLP Compliance Monitoring Authority of India and by the Food and Consumer Product Safety Authority of the OECD. All studies described in this study were audited and verified by the laboratory's Quality Assurance Unit.

### Test material

The test material, micronized PEA (particle size 0.5–10 *μ*m; purity 99%; microPEA), was manufactured by Epitech Group Srl, Milano Italy, supplied by Prismic, and met all specifications as outlined in the required Certificate of Analysis. The term microPEA in this paper includes both micronized and ultramicronized PEA, as the particle size distribution of 0.5‐10 µm includes both fractions. The analytical method was validated in a GLP study which also verified the homogeneity of microPEA in the dosing matrix used in the animal studies as well as its stability over the period from preparation to dosing (Intox, 2015e). The test article was characterized by the sponsor who provided an authorized “Certificate of Analysis” for each study described in this study.

### In vitro studies

#### Metabolic activation

As suggested by Maron and Ames (1983), some degree of mammalian metabolism was introduced into the in vitro test systems by using a fraction of rat liver, referred to as S9. S9 was prepared on site at Intox after inducing metabolizing enzymes by injection of rats with phenobarbitone and *β*‐naphthoflavone (OECD, 1997).

### Bacterial reverse mutation test

The Salmonella mammalian microsome reverse mutation test protocol conformed to the appropriate OECD Guideline (OECD, 1997), which is based on the ‘Ames’ test as described by Maron and Ames (1983), and was found to be compliant with GLP (Intox, 2015a).

#### Bacterial strains

The tester strains TA1535, TA97a, TA98, TA100, and TA102, as recommended (OECD, 1997), were obtained from Moltox of Boone, North Carolina. Their identity and characteristics were verified prior to testing (Intox, 2015a).

#### Preliminary range‐finding experiments

MicroPEA was found to be uniformly suspended in Pluronic F68 at the concentration of 50 mg/mL. It was shown to precipitate when incorporated into agar plates at the levels of 4000 and 5000 but not at 3000 *μ*g/plate, chosen as the highest test dose as recommended by OECD (1997). The doses of 3000, 2000, 1000, 500, and 250 *μ*g/plate were selected to evaluate toxicity in strain TA100 with the presence and absence of S9. Slight cytotoxicity was found at 3000 *μ*g/plate (‐S9), the maximum dose for both the plate incorporation test and preincubation assay (Intox, 2015a).

#### Controls

Negative, including blank sterility controls, and positive controls were included in the testing of microPEA, both in the presence and absence of S9 to verify spontaneous mutation frequencies and strain‐specific responses to known mutagens. Positive control compounds without S9 mix were as follows: sodium azide (Sigma Aldrich St. Louis, MO, USA) for TA1535, ICR 191 (Sigma Aldrich) for TA97a; 3‐methylmethane sulfonate (Sigma Aldrich) for TA100 and TA102; and, 4‐nitroquinoline‐N‐oxide (Acros Organics, Geel, Belgium) for TA98. Positive controls with S9 mix were as follows: 2‐aminoanthracene (Sigma Aldrich) for TA1535; 2‐aminofluorene (Sigma Aldrich) for TA97a, TA98, and TA100; and, danthron (Sigma Aldrich) for TA102.

#### Media

Test strains were grown overnight in Oxoid nutrient broth No. 2, combined with test chemical and S9 in soft overlay agar containing 0.6% agar, 0.5% NaCl, and 0.05 mmol/L histidine–biotin, then plated on agar (2%) containing Vogel–Bonner minimal medium E and 2% glucose, as previously described (Maron and Ames 1983).

#### Test method

Both plate incorporation and preincubation methods (OECD, 1997) were employed, both in the absence and presence of S9. For preincubation, the treatment mixtures were incubated at 37C for 20 min prior to plating. Test plates in triplicate were incubated at 37°C for 68–72 h at which time revertant colonies were counted and the plates were examined for extent of background growth as an indicator of cytotoxicity.

### In vitro mammalian cell micronucleus test

The test protocol conformed to the appropriate OECD Guideline (OECD, 2014) and was performed according to GLP (Intox, 2015b), using cultured lymphocytes from healthy human volunteers.

#### Preliminary range‐finding experiments

MicroPEA was found to be sufficiently suspended in Pluronic F68 at the concentration 1000 *μ*g/mL, with minimum precipitation and no cytotoxicity as measured by the cytokinesis‐block proliferation index (CBPI). Based on these results, the tests were performed with 250, 500, and 1000 *μ*g/mL (Intox, 2015b).

#### Controls

Controls included negative (NaCl) and vehicle (Pluronic F68) controls, ±S9. Positive controls –S9 included the aneugenic compound vinblastine, and clastogenic agent mitomycin C. Cyclophosphamide was the positive control to show that the S9 metabolic fraction was active.

#### Test method (OECD, 2014)

Experiments 1 and 2 were conducted in the absence of S9, with exposure of the cells to microPEA for 3 h and 24 h, respectively. Experiment 3 involved exposure for 3 h in the presence of S9. All experiments were conducted with duplicate cultures of cells, after growing exponentially for 48 h from culture initiation. Cytochalasin B was added after termination of treatment in Experiments 1 and 3, and added with treatment in Experiment 2. Cell harvesting was done 72 h after culture initiation, slides were prepared, and the cells were stained with 5% Giemsa. Microscopic examination of 2000 cells determined the percent incidence of micronuclei in binucleated cells (BNCs).

### In vivo studies

#### Delivery of test article

Preliminary tests found that microPEA was suitable for oral gavage by suspension in water with Tween 80 (0.5% w/v) as a surfactant and carboxymethyl cellulose (1% w/v) as a suspending agent (Intox, 2015c,d, 2016). Analyses performed to verify the concentrations of microPEA in dosing formulations in the 90‐day study showed that they were within an acceptable range compared to their respective nominal concentrations (Intox, 2016).

### Acute oral toxicity

#### Study design

MicroPEA was tested, in accordance with GLP, in the female rat to determine its potential to cause acute lethality (Intox, 2015c), using the “Up and Down Procedure” described in Guideline 425 of OECD (2008a). To minimize the number of animals, the up and down procedure adopted by OECD (2008a) for acute toxicity testing strives to use a maximum of five animals, starting with a single dose to a single rat. After a period of observation, the dose may or may not be adjusted up or down before dosing the next animal. As microPEA is not expected to have potent toxicity, the intent of this study was to test at the maximum dose of 2000 mg/kg bw. Thus, this test is characterized as a limit test, using Sprague‐Dawley rats (Vivo Bio Tech Ltd, Telangana, India).

#### Test method (OECD, 2008a)

The rat is the preferred rodent species for this test, and the female is recommended for testing since in cases where differences are found between male and female rats, the female is usually the more sensitive sex. Administration of the test material is by gavage. A limit test is suggested for materials suspected to be relatively nontoxic, as is the case for microPEA. A stepwise progression begins with dosing one animal with the limit dose. The main LD50 study is conducted if the animal dies within 48 h, but if not, then four additional animals are dosed sequentially at a minimum of 48 h intervals. The resulting LD50 is >2000 mg/kg bw when three or more animals survive after 14 days. Results to be recorded include body weight, observations of any sign(s) of toxicity, and any gross pathological changes.

### 14‐day study

#### Study design

Sprague‐Dawley rats (Vivo Bio Tech Ltd), age 6–7 weeks, in groups of five of each sex, were gavaged with a vehicle control and with microPEA at doses of 100, 300, and 1000 mg/kg bw/day (Intox, 2015c). This study was conducted according to GLP.

#### Test method (OECD, 2008b)

This study, intended to be a dose‐ranging study to identify doses to be used in a subsequent 90‐day study, was based on recommendations in the OECD Guideline for the Repeated Dose 28‐day Oral Toxicity Study in Rodents (OECD, 2008b). The preferred species is the rat, and administration by gavage is a recommended method of dose delivery. Ten animals, five of each sex, are used for each dose delivered daily for the duration of the study. Three dose levels set at two to four times intervals plus control are used, with 1000 mg/kg bw considered a maximum dose. Dosage is daily for the duration of the study with daily observation for mortality and clinical signs. Body weight and food consumption should be recorded weekly at a minimum. Samples for hematology and clinical chemistry analysis are taken just prior to sacrifice, and gross necropsy was performed, recording any gross pathological observations and organ weights.

### 90‐day study

#### Study design

Rats (Sprague‐Dawley, as above) were divided into six groups. Four groups of 20 (10 males and 10 females) were treated for 90 days by oral gavage with the vehicle control (G1), the low dose of 250 mg/kg bw/day (G2), the mid dose of 500 mg/kg bw/day (G3), or the high dose of 1000 mg/kg bw/day (G4). An additional 10 animals (five males and five females) in the control [G1(R)] and in the high‐dose [G4(R)] groups were allowed to recover for an additional 28 days (Intox, 2016). This study was conducted according to GLP.

#### Test method (OECD, 1998)

The rat is the preferred rodent according to the OECD Guideline for the Repeated Dose 90‐Day Oral Toxicity Study in Rodents (OECD, 1998), and dosing every day for 90 consecutive days should begin as soon as possible after weaning. At least 20 animals (10 males and 10 females) are used for each dose level and control. It is recommended that groups of 10 control animals (five males and five females) and 10 animals (five males and five females) treated at the highest dose be maintained after the treatment period to see whether treated animals recover from toxic effects or if they persist. Three dose levels set at two to four times intervals plus control are used, with 1000 mg/kg bw considered a maximum dose. Dosage is daily for the duration of the study with twice daily observation for mortality and weekly for clinical signs. Ophthalmological examinations are performed before study initiation and at study termination. Neurological parameters include qualitative and quantitative assessment of sensory reactivity, grip strength, motor activity, frequency of urination, defecation, rearing, and landing foot splay. Body weight and food consumption are recorded weekly at a minimum. Samples for hematology and clinical chemistry analysis are taken just prior to sacrifice. Hematological parameters include hematocrit, hemoglobin concentration, erythrocyte count, total and differential leukocyte count, platelet count, and blood clotting time. Clinical chemistry determinations include sodium, potassium, glucose, total cholesterol, urea, blood urea nitrogen, creatinine, total protein and albumin, and two or more enzymes that indicate hepatocellular effects. Urine samples taken during the last week before sacrifice are examined for appearance, volume, osmolality or specific gravity, pH, protein, glucose, and presence of blood or blood cells. At study termination, gross necropsy is performed including complete external body examination and organ weights recorded for liver, kidneys, adrenals, testes, epididymides, uterus, ovaries thymus, spleen, brain, and heart. Histopathological examination is performed on tissue samples from: all gross lesions, representative brain regions, spinal cord at three levels, pituitary, thyroid, parathyroid, thymus, esophagus, salivary glands, stomach, small and large intestines (including Peyer's patches), liver, pancreas, kidneys, adrenals, spleen, heart, trachea, lungs, aorta, gonads, uterus, accessory sex organs, female mammary gland, prostate, urinary bladder, lymph nodes, peripheral nerve, bone marrow section, skin, and eyes if changes were observed upon examination. Tissue samples are fixed in 10% neutral buffered formalin before embedding in paraffin wax. Sections of 5 *μ*m thickness are stained with hematoxylin and eosin for microscopic examination.

The results were analyzed statistically with IBM SPSS Statistical Software (version 23). Following assessment of homogeneity, using Levene's test, of body weight, food consumption, hematology, clinical chemistry, organ weight, and neurological examination data, different groups were subjected to one‐way analysis of variance (ANOVA). Comparisons between treated and control groups were analyzed by *t* tests with variance evaluated at the 5% level of significance.

## Results

### Ames test

Results from triplicate cultures in Experiment 1 using the plate incorporation test, and in Experiment 2 using the preincubation assay show that microPEA did not induce a dose‐dependent or 2‐fold increase in revertant colonies at doses of 30, 90, 300, 900, and 3000 *μ*g/plate, without and with S9 for metabolic activation (Tables 1 and 2). Neither were there any cytotoxic effects. All negative and positive control values were within expected and normal ranges. Thus, it is concluded that microPEA is not mutagenic in the Ames test (Intox, 2015a).

**Table 1:** Summary data on histidine revertant colonies (plate incorporation method)

Treatment	Concentration		TA1535		TA97a		TA98		TA100		TA102	
	(*μ*g/plate)	S9	Mean	±SD	Mean	±SD	Mean	±SD	Mean	±SD	Mean	±SD
Micronized Palmitoylethanolamide (particle size 0.5–10 *μ*m)	3000	−	17.67	1.53	111.33	13.32	18.33	3.06	120.00	5.29	264.00	10.58
	900		20.00	4.36	128.67	8.33	22.00	1.00	124.00	5.29	230.67	16.65
	300		16.00	2.65	146.00	4.00	29.67	5.03	124.67	2.31	246.67	16.65
	90		9.67	3.06	102.67	15.53	32.67	3.79	128.00	2.00	265.33	10.07
	30		16.67	3.79	92.67	3.06	30.67	6.66	124.00	5.29	272.00	28.00
Negative control	0	+	6.00	2.65	115.33	15.14	19.67	3.06	130.00	6.93	258.67	18.04
Vehicle control (pluronic F68)	100 *μ*L		13.33	3.51	97.33	8.08	21.33	2.52	124.00	5.29	256.00	10.58
Micronized palmitoylethanolamide (particle size 0.5–10 *μ*m)	3000		17.00	1.00	112.67	12.22	32.33	1.53	125.33	3.06	254.67	12.22
	900		17.67	3.06	106.67	9.02	36.67	2.08	129.33	7.02	274.67	18.04
	300		22.33	2.08	109.33	7.02	41.33	1.53	120.00	5.29	260.00	16.00
	90		25.67	5.86	107.33	11.02	26.67	2.08	128.00	5.29	256.00	17.44
	30		18.00	2.65	122.67	1.15	26.00	4.36	136.00	2.00	250.67	6.11
Negative control	0		10.67	3.79	100.00	16.37	29.00	4.36	131.00	6.11	258.67	16.17
Vehicle control (Pluronic F68)	100 *μ*L		13.00	1.00	105.33	9.45	26.67	2.08	128.67	8.33	258.67	6.11
Positive controls												
Sodium azide	2	−	261.33	40.07	–	–	–	–	–	–	–	–
ICR 191	1		–	–	674.67	153.74	–	–	–	–	–	–
4‐Nitroquinoline‐N‐oxide	0.5		–	–	–	–	653.33	106.53	–	–	–	–
3‐Methylmethane sulfonate	1 *μ*L		–	–	–	–	–	–	1272.00	115.38	1360.00	21.17
2‐Aminoanthracene	10	+	256.00	18.33	–	–	–	–	–	–	–	–
2‐Aminofluorene	20		–	–	743.00	41.80	1445.33	41.05	1432.00	127.00	–	–
Danthron	30		–	–	–	–	–	–	–	–	1664.00	170.27

S9: (−) Without S9, (+) With S9.

**Table 2:** Summary data on histidine revertant colonies (preincubation method)

Treatment	Concentration		TA1535		TA97a		TA98		TA100		TA102	
	(*μ*g/plate)	S9	Mean	±SD	Mean	±SD	Mean	±SD	Mean	±SD	Mean	±SD
Micronized palmitoylethanolamide (particle size 0.5–10 *μ*m)	3000	−	14.00	3.61	110.67	13.61	38.33	3.79	138.00	10.58	234.67	22.03
	900		10.33	1.53	129.33	8.08	28.33	8.02	154.00	5.29	289.33	28.10
	300		11.00	1.73	111.33	6.11	24.00	2.65	146.00	14.42	328.00	30.20
	90		17.33	5.51	124.00	5.29	24.67	1.15	154.67	6.43	280.00	26.23
	30		13.33	1.15	125.33	3.06	30.00	2.65	148.00	2.00	264.00	10.58
Negative control	0		14.33	3.51	101.33	4.16	25.33	6.81	128.00	5.29	282.67	30.02
Vehicle control (Pluronic F68)	100 *μ*L		19.00	4.36	108.00	5.29	38.67	4.51	129.33	3.06	250.67	8.33
Micronized palmitoylethanolamide (particle size 0.5–10 *μ*m)	3000	+	11.33	5.86	114.67	6.43	39.33	1.53	158.00	24.33	256.00	4.00
	900		7.33	2.52	124.00	3.46	42.00	2.65	136.00	12.17	245.33	6.11
	300		16.67	3.79	128.00	5.29	40.00	2.65	146.00	2.00	253.33	8.33
	90		11.67	0.58	104.67	4.16	37.67	4.73	121.33	8.33	249.33	8.33
	30		15.00	4.58	110.67	6.43	25.67	3.06	124.67	7.57	248.00	10.58
Negative control	0		15.67	2.52	96.67	10.07	22.00	7.94	117.33	6.43	282.67	38.85
Vehicle control (Pluronic F68)	100 *μ*L		25.00	2.65	108.00	5.29	31.67	3.06	158.67	34.08	254.67	9.24
Positive controls												
Sodium azide	2	−	650.67	57.87	–	–	–	–	–	–	–	–
ICR 191	1		–	–	765.33	60.04	–	–	–	–	–	–
4‐Nitroquinoline‐N‐oxide	0.5		–	–	–	–	1421.33	124.02	–	–	–	–
3‐Methylmethane sulfonate	1 *μ*L		–	–	–	–	–	–	770.67	56.19	1533.33	53.27
2‐Aminoanthracene	10	+	693.33	113.23	–	–	–	–	–	–	–	–
2‐Aminofluorene	20		–	–	564.00	90.60	608.00	152.63	518.67	232.91	–	–
Danthron	30		–	–	–	–	–	–	–	–	1210.67	162.45

S9: (−) Without S9, (+) With S9.

### In vitro mammalian cell micronucleus test

MicroPEA did not induce a biologically significant, dose‐related increase in the percent incidence of micronuclei in binucleated cells (BNCs) at any dose in any of the three experiments, either in the absence or presence of S9 for mammalian metabolism (Tables 3, 4, 5). In addition, microPEA did not produce cytotoxicity as assessed by the cytokinesis‐block proliferation index (CBPI) in either the preliminary cytotoxicity screen or in the three experiments as described above (Intox, 2015b).

**Table 3:** Summary of incidence of micronucleated BNCs and cytotoxicity

Cultured lymphocytes treated for 3 h without metabolic activation – Experiment no. 1						
Test/control article & dose (*μ*g/mL)	Dose *μ*g/mL	No. of cells analyzed	No. of BNC with MN	% of BNC with MN	CBPI	% Cytostasis
Negative control 0.9% Saline w/v	–	2008	3	0.15	1.87	–
Vehicle control pluronic F68	–	2005	5	0.25	1.85	–
Positive control MMC	0.8	2000	31	1.55^a^	1.55	35.65
Positive control VBL	0.08	2000	31	1.55^a^	1.56	34.38
Micronized palmitoylethanolamide (particle size 0.5–10 *μ*m)	1000	2000	6	0.30	1.71	16.95
	500	2013	4	0.20	1.75	11.86
	250	2000	5	0.25	1.74	13.27

a *P* < 0.05.

**Table 4:** Summary of incidence of micronucleated BNCs and cytotoxicity

Cultured lymphocytes treated for 24 h without metabolic activation – Experiment no. 2						
Test/control article & dose (*μ*g/mL)	Dose *μ*g/mL	No. of cells analyzed	No. of BNC with MN	% of BNC with MN	CBPI	% Cytostasis
Negative control 0.9% saline w/v	–	2006	3	0.15	1.76	–
Vehicle control pluronic F68	–	2010	4	0.20	1.77	–
Positive control MMC	0.8	2000	34	1.70^a^	1.58	24.51
Positive control VBL	0.08	2000	29	1.45^a^	1.59	23.97
Micronized palmitoylethanolamide (particle size 0.5–10 *μ*m)	1000	2000	7	0.35	1.62	19.72
	500	2000	6	0.30	1.64	17.40
	250	2000	5	0.25	1.75	3.66

a *P* < 0.05.

**Table 5:** Summary of incidence of micronucleated BNCs and cytotoxicity

Cultured lymphocytes treated for 3 h with metabolic activation – Experiment no. 3						
Test/control article & dose (*μ*g/mL)	Dose *μ*g/mL	No. of cells analyzed	No. of BNC with MN	% of BNC with MN	CBPI	% Cytostasis
Negative control 0.9% saline w/v	–	2007	2	0.10	1.79	–
Vehicle control pluronic F68	–	2007	4	0.20	1.78	–
Positive control CPM	6.25	2000	36	1.80^a^	1.68	13.82
Micronized palmitoylethanolamide (particle size 0.5–10 *μ*m)	1000	2006	2	0.10	1.74	5.38
	500	2021	3	0.15	1.76	3.35
	250	2000	4	0.20	1.75	4.20

a *P* < 0.05.

### Acute oral toxicity study

MicroPEA did not induce any mortality at the limit dose of 2000 mg/kg bw or abnormal clinical signs in any of the five test animals observed at 10 and 30 min, 1, 2, and 4 h, and thereafter daily until day 15 postdosing. Each animal was subjected to gross necropsy, and no gross pathological abnormalities were found. Microscopic pathology was not conducted in the absence of any gross pathological changes. The LD50 was reported to be >2000 mg/kg bw (Intox, 2015c).

### 14‐day oral toxicity study

No incidence of clinical signs or death was found following 14 days of treatment with microPEA at 0, 100, 300, or 1000 mg/kg bw/day. Furthermore, no adverse effects were observed on body weight gain, food consumption, hematological parameters, clinical chemistry, gross pathology, or on absolute or relative organ weights (data not shown). Thus, the No Observed Effect Level (NOEL) from this study is >1000 mg/kg bw/day (Intox, 2015c).

### 90‐day oral toxicity study

No treatment‐related adverse effects were found in this subchronic study, and the NOEL is considered to be the highest dose level of 1000 mg/kg bw/d. There were some incidental findings, described below, that are concluded to be unrelated to treatment and of no toxicological significance.

Survival was 100%, for both male and female rats, in all control, treatment, and recovery groups. No clinical signs were found in any group other than respiratory rales in one control male (day 84 until termination at day 91) and one treated, mid‐dose (500 mg/kg bw/d) male from day 80 until day 91; these observations were incidental, not dose‐related, and of no toxicological significance. No ophthalmological effects were found in any control or treated animal, either before or after dosing, and neurological observations made during the 12th week were the same in control and treated, male and female rats. Similarly, urine samples taken on day 88 of the study showed no significant differences in urinalysis parameters between high‐dose males and females compared to their respective control groups.

There was no difference in average body weights between the control and any of the three treatment groups after 90 days (Table 6). The average weight of male animals in the high treatment recovery group [G4(R)], however, was less than the average of males in the control recovery group [G1(R)], a difference that was statistically significant (*P *<* *0.05). Although this comparison makes it appear that the G4(R) male animals lost weight between Day 90 and Day 97 while G1(R) control males gained weight over that week, this was not the case. Further analysis reveals that the subset of 5 G4(R) male animals was smaller from early in the study and gained weight as normally as the other 10 G4 males over the full course of the treatment and recovery periods. The individual animal data show that the subset of 5 G4(R) treated males weighed slightly less than the G1(R) control males from Day 7 of the study, but not enough to skew the combined average weights of all 15 treated [G4/G4(R)] male animals compared to the combined average weights of all 15 male control [G1/G1(R)] animals. The average body weights for both combined [G1/G1(R)] and combined [G4/Gr(R)] males at day 90 are identical (475 g). Although the average weights of the 15 animals in the combined groups are the same, there is a difference in average weights between the subsets of five G1(R) males and five G4(R) males at day 90 (484 and 437 g, respectively). This difference between these subsets of five animals is not a result of an adverse effect on weight gain which was normal for all test animals. Furthermore, weekly measurements of food consumption showed no differences between treated and control groups over the course of the 90‐day study and recovery period. Based on these observations and the analysis described above, it is concluded that consumption of microPEA did not have an adverse effect on body weight or body weight gain.

**Table 6:** Summary of body weights (g)

Male rats																				
Group	Dose mg/kg/day		Study days																	
			1	7	14	21	28	35	42	49	56	63	70	77	84	90	97	104	111	118
Vehicle control: analytical grade water with Tween 80 (about 0.5% w/v) and carboxymethyl cellulose (1% w/v)																				
G1 & G1 (R)	0	Mean	253.13	286.60	315.20	341.67	371.33	383.53	402.13	419.93	432.20	445.27	457.07	468.53	473.47	474.67	490.20	494.40	498.00	500.80
		±SD	14.16	17.26	22.68	23.88	23.44	25.58	29.44	32.04	33.13	34.01	33.71	36.10	37.75	41.83	35.15	36.21	35.12	35.37
		*n*	15	15	15	15	15	15	15	15	15	15	15	15	15	15	5	5	5	5
Test article: micronized palmitoylethanolamide (particle size 0.5–10 *μ*m)																				
G2*n* = 10	250	Mean	254.10	287.80	312.60	337.60	358.60	371.10	390.70	408.00	419.90	432.20	439.70	449.00	454.30	459.30	–	–	–	–
		±SD	15.01	14.43	16.32	17.63	17.21	17.63	18.49	23.95	26.87	25.40	26.22	25.77	25.92	26.07	–	–	–	–
G3*n* = 10	500	Mean	252.90	292.40	325.10^a^	349.00	372.90	386.50	405.60	422.10	436.50	449.40	456.70	469.80	472.20	475.30	–	–	–	–
		±SD	17.70	15.37	14.23	15.08	20.58	21.40	24.61	26.48	28.78	30.03	29.41	27.45	23.18	22.55	–	–	–	–
G4 & G4 (R)	1000	Mean	253.87	290.27	317.20	354.13	377.47	390.07	406.33	421.73	433.80	443.67	453.07	463.27	470.40	475.00	439.80^a^	445.40^a^	448.60^a^	451.20^a^
		±SD	12.89	16.39	28.55	25.14	27.69	31.47	32.04	34.07	35.46	35.66	35.62	38.62	40.44	42.03	16.62	17.17	16.91	17.54
		*n*	15	15	15	15	15	15	15	15	15	15	15	15	15	15	5	5	5	5

Female rats																				
Group	Dose mg/kg/day		Study days																	
			1	7	14	21	28	35	42	49	56	63	70	77	84	90	97	104	111	118
Vehicle control: analytical grade water with Tween 80 (about 0.5% w/v) and carboxymethyl cellulose (1% w/v)																				
G1 & G1 (R)	0	Mean	194.20	210.60	222.73	238.60	247.60	255.53	263.67	268.67	271.93	274.47	281.20	285.20	289.07	292.07	295.80	297.60	299.60	300.40
		±SD	7.63	8.42	9.32	13.34	12.19	13.64	15.14	15.74	16.37	17.15	19.42	20.15	21.05	21.26	30.86	30.64	30.65	30.75
		*n*	15	15	15	15	15	15	15	15	15	15	15	15	15	15	5	5	5	5
Test article: micronized palmitoylethanolamide (particle size 0.5–10 *μ*m)																				
G2*n* = 10	250	Mean	194.50	212.30	224.20	242.60	253.60	260.00	267.80	272.60	277.50	280.90	285.00	290.70	294.20	297.30	–	–	–	–
		±SD	12.11	13.69	13.09	13.44	16.42	16.83	16.59	17.94	19.61	20.58	20.55	20.90	20.75	21.11	–	–	–	–
G3*n* = 10	500	Mean	193.40	211.40	224.80	237.30	246.40	254.00	262.80	264.50	272.10	274.90	281.00	286.30	290.10	292.40	–	–	–	–
		±SD	8.33	11.57	9.75	9.36	9.31	10.04	10.85	9.66	12.90	13.59	14.45	14.19	14.69	14.77	–	–	–	–
G4 & G4 (R)	1000	Mean	193.60	211.40	225.73	236.20	245.40	253.27	262.07	266.27	270.73	273.47	278.33	283.13	289.53	291.80	286.80	289.40	291.40	292.20
		±SD	7.31	9.15	11.41	11.87	11.69	11.96	13.07	14.72	14.35	14.52	15.69	15.68	17.22	17.90	10.18	8.56	8.05	7.66
		*n*	15	15	15	15	15	15	15	15	15	15	15	15	15	15	5	5	5	5

a Significantly different from control group (*P* < 0.05).

Table 7 shows a summary of hematology data for all animals, male and female, control, treated, and recovery. The results are normal with no differences between control and treated groups except for elevated total WBC counts for the high‐dose group at the end of the recovery period. Although statistically higher than concurrent controls, the value 10.08 × 10^3^/cm^2^ is well within the historical range (8.3 to 18.5 × 10^3^/cm^2^) for control rats in 90‐day studies at the test facility. Furthermore, no correlating adverse effects were observed in this test group with regard to clinical signs, blood chemistry, organ weights, and gross or microscopic pathology, indicating no toxicological significance for this incidental finding.

**Table 7:** Summary of hematology data

Male rats																			
End of treatment period (Day 91)																			
Group	Dose (mg/kg/day)		Hb (g/dL)	PCV (%)	Total RBC (×10^6^/cmm)	RBC indices			Total WBC (×10^3^/cmm)	Differential WBC count (%)					Platelets (×10^3^/cmm)	Prothrombin time (s)	APTT (s)	Reticulocyte count (%)	General blood picture
						MCH (pg)	MCV (fL)	MCHC (g/dL)		N	L	M	E	B					
Vehicle control: analytical grade water with Tween 80 (about 0.5% w/v) and carboxymethyl cellulose (1% w/v)																			
G1*n* = 10	0	Mean	15.23	41.82	8.57	17.81	48.87	36.45	5.79	29.71	67.00	0.898	2.368	0.003	890.50	16.05	15.29	1.77	NAD
		±SD	0.68	2.43	0.62	0.91	2.08	0.74	1.60	13.19	12.49	0.402	0.882	0.010	220.47	1.10	2.33	0.43	–
Test article: micronized palmitoylethanolamide (particle size 0.5–10 *μ*m)																			
G2*n* = 10	250	Mean	15.21	41.60	8.54	17.85	48.86	36.57	5.85	31.34	65.39	0.570	2.700	0.000	890.10	15.74	15.84	1.83	NAD
		±SD	0.68	1.99	0.51	1.15	3.64	0.48	1.05	12.86	11.74	0.354	1.483	0.000	290.86	0.65	2.06	0.41	–
G3*n* = 10	500	Mean	15.03	41.31	8.55	17.59	48.34	36.41	6.17	35.28	61.26	0.660	2.759	0.000	865.80	16.46	15.29	2.22	NAD
		±SD	0.64	2.15	0.40	0.71	2.39	0.83	1.06	7.58	7.09	0.289	1.286	0.000	269.06	1.40	2.37	0.59	–
G4*n* = 10	1000	Mean	15.59	42.79	8.68	18.02	49.46	36.44	6.48	29.45	67.38	0.719	2.436	0.000	846.20	15.85	15.66	2.23	NAD
		±SD	0.54	1.64	0.55	1.16	3.03	0.45	1.48	3.81	3.71	0.443	0.667	0.000	217.04	1.00	2.28	0.70	–

Female rats																			
End of treatment period (Day 91)																			
Group	Dose (mg/kg/day)		Hb (g/dL)	PCV (%)	Total RBC (×10^6^/cmm)	RBC indices			Total WBC (×10^3^/cmm)	Differential WBC count (%)					Platelets (×10^3^/cmm)	Prothrombin time (s)	APTT (s)	Reticulocyte count (%)	General blood picture
						MCH (pg)	MCV (fL)	MCHC (g/dL)		N	L	M	E	B					
Vehicle Control: Analytical grade water with Tween 80 (about 0.5% w/v) and carboxymethyl cellulose (1% w/v)																			
G1*n* = 10	0	Mean	14.68	40.44	7.72	19.06	52.48	36.32	3.55	20.83	74.46	0.795	3.922	0.004	748.20	13.68	12.62	2.93	NAD
		±SD	0.50	1.65	0.45	0.81	1.63	0.76	1.78	5.91	5.75	0.244	0.906	0.014	331.40	1.24	1.61	0.77	–
Test article: micronized palmitoylethanolamide (particle size 0.5–10 *μ*m)																			
G2n = 10	250	Mean	14.57	40.23	7.79	18.76	51.81	36.23	5.59	26.36	68.31	2.863	2.372	0.129	879.20	14.55	13.61	2.76	NAD
		±SD	0.86	2.59	0.49	1.45	4.29	0.64	2.66	14.15	15.39	3.100	1.273	0.255	270.69	2.20	1.72	0.73	–
G3*n* = 10	500	Mean	14.99	42.15	7.91	18.97	53.33	35.57	3.68	28.64	67.20	0.840	3.304	0.006	823.30	14.97	13.45	2.53	NAD
		±SD	0.57	1.65	0.38	0.43	1.21	0.59	1.00	9.17	8.60	0.275	1.856	0.013	365.14	1.62	1.49	0.71	–
G4*n* = 10	1000	Mean	15.10	41.96	7.94	19.03	52.87	36.00	4.42	27.48	67.40	2.140	2.984	0.003	802.30	12.86	14.23	2.66	NAD
		±SD	0.55	1.92	0.38	0.49	1.80	0.64	1.44	9.67	10.44	2.663	0.792	0.009	254.75	1.25	1.03	1.06	–

Male rats																			
Termination of recovery period (Day 119)																			
Group	Dose (mg/kg/day)		Hb (g/dL)	PCV (%)	Total RBC (×10^6^/cmm)	RBC indices			Total WBC (×10^3^/cmm)	Differential WBC count (%)					Platelets (×10^3^/cmm)	Prothrombin time (s)	APTT (s)	Reticulocyte count (%)	General blood picture
						MCH (pg)	MCV (fL)	MCHC (g/dL)		N	L	M	E	B					
Vehicle control: analytical grade water with Tween 80 (about 0.5% w/v) and carboxymethyl cellulose (1% w/v)																			
G1 (R)*n* = 5	0	Mean	15.20	42.66	8.75	17.38	48.78	35.62	6.56	30.32	66.96	0.697	2.024	0.000	931.20	18.00	14.86	1.91	NAD
		±SD	0.80	2.01	0.41	0.32	1.02	0.27	1.17	6.25	5.66	0.380	0.766	0.000	189.98	1.26	1.27	0.47	–
Test article: micronized palmitoylethanolamide (particle size 0.5–10 *μ*m)																			
G4 (R)*n* = 5	1000	Mean	13.70	39.32	7.20	19.39	55.98	34.80	10.08^a^	25.64	68.18	3.718	2.456	0.022	888.40	18.62	14.92	2.13	NAD
		±SD	1.94	4.93	1.62	2.47	9.31	1.24	2.12	7.70	11.26	5.311	0.697	0.041	60.01	0.74	1.80	0.32	–

Female rats																			
Termination of recovery period (Day 119)																			
Group	Dose (mg/kg/day)		Hb (g/dL)	PCV (%)	Total RBC (×10^6^/cmm)	RBC indices			Total WBC (×10^3^/cmm)	Differential WBC count (%)					Platelets (×10^3^/cmm)	Prothrombin time (s)	APTT (s)	Reticulocyte count (%)	General blood picture
						MCH (pg)	MCV (fL)	MCHC (g/dL)		N	L	M	E	B					
Vehicle control: analytical grade water with Tween 80 (about 0.5% w/v) and carboxymethyl cellulose (1% w/v)																			
G1 (R)*n* = 5	0	Mean	14.88	40.34	7.93	18.80	50.95	36.90	4.07	31.76	64.72	0.990	2.434	0.068	785.00	17.22	11.94	1.67	NAD
		±SD	0.53	1.84	0.52	0.74	1.58	0.46	1.32	9.56	9.51	0.365	0.884	0.132	233.93	0.42	1.64	0.34	–
Test article: micronized palmitoylethanolamide (particle size 0.5–10 *μ*m)																			
G4 (R)*n* = 5	1000	Mean	14.98	40.36	7.82	19.23	51.80	37.12	8.01	23.14	73.76	0.466	2.618	0.033	906.60	17.98	12.08	2.70	NAD
		±SD	0.24	1.00	0.60	1.41	3.64	0.53	3.75	5.95	5.70	0.341	0.890	0.063	89.16	1.91	0.51	1.08	–

Hemoglobin, hematocrit (PCV), total erythrocyte count (total RBC), total leukocyte count (total WBC), differential leukocyte (WBC) counts, erythrocyte indices, namely, mean corpuscular volume (MCV), mean corpuscular hemoglobin (MCH), mean corpuscular hemoglobin concentration (MCHC), total platelet count, reticulocyte count, and coagulation parameters namely. prothrombin time (PT) and activated partial thromboplastin time (APTT).

a Significantly different from control group (*P* < 0.05).

Clinical chemistry findings are shown in Table 8. A few isolated measurements were statistically different from controls, but are considered to be incidental and without biological significance due to lack of any dose dependency as well as their values falling within ranges of the laboratory's historical controls.

**Table 8:** Summary of clinical chemistry data

Male rats																					
End of treatment period (Day 91)																					
Group	Dose (mg/kg/day)		TP (g/dL)	ALB (g/dL)	ALT (IU/L)	AST (IU/L)	ALP (IU/L)	TBIL (mg/dL)	GLU (mg/dL)	CHOL (mg/dL)	BUN (mg/dL)	UREA (mg/dL)	CRE (mg/dL)	GGT (IU/L)	TG (mg/dL)	Na (mmol/L)	K (mmol/L)	Ca (mg/dL)	PHO (mg/dL)	Globulin (g/dL)	A/G Ratio (g/dL)
Vehicle: analytical grade water with Tween 80 (about 0.5% w/v) and carboxymethyl cellulose (1% w/v)																					
G1*n* = 10	0	Mean	6.70	1.14	38.50	73.80	98.90	0.10	108.40	99.30	15.70	22.61	0.40	4.40	81.40	151.50	5.12	9.66	6.80	5.56	0.21
		±SD	0.33	0.12	10.22	9.90	13.92	0.00	26.73	13.47	2.98	4.30	0.07	1.07	32.85	4.43	0.29	0.25	0.67	0.29	0.02
Test article: micronized palmitoylethanolmide (particle size 0.5–10 *μ*m)																					
G2*n* = 10	250	Mean	6.93	1.18	43.80	120.80	99.00	0.09	103.80	92.70	14.30	20.59	0.40	3.90	95.50	151.00	5.10	9.82	6.79	5.75	0.21
		±SD	0.25	0.28	19.31	45.94	46.48	0.03	9.38	18.11	1.95	2.80	0.08	0.88	35.68	3.13	0.22	0.22	0.53	0.39	0.06
G3*n* = 10	500	Mean	6.78	1.05	56.60	105.30^a^	122.60	0.10	111.50	86.70	15.90	22.90	0.42	4.60	90.50	149.50	5.26	9.37	7.58^a^	5.73	0.19
		±SD	0.31	0.31	31.02	25.74	45.41	0.00	16.69	14.00	2.23	3.22	0.04	1.26	39.93	4.43	0.30	0.43	0.75	0.37	0.06
G4*n* = 10	1000	Mean	7.19^a^	1.33	45.50	90.60	97.80	0.09	108.00	98.80	15.60	22.46	0.41	4.50	103.30	152.00	5.20	9.66	6.86	5.86	0.23
		±SD	0.28	0.08	15.28	18.46	8.77	0.03	4.37	17.40	2.22	3.20	0.09	0.71	20.62	1.94	0.30	0.23	0.44	0.30	0.02

Female rats																						
End of treatment period (Day 91)																						
Group	Dose (mg/kg/day)			TP (g/dL)	ALB (g/dL)	ALT (IU/L)	AST (IU/L)	ALP (IU/L)	TBIL (mg/dL)	GLU (mg/dL)	CHOL (mg/dL)	BUN (mg/dL)	UREA (mg/dL)	CRE (mg/dL)	GGT (IU/L)	TG (mg/dL)	Na (mmol/L)	K (mmol/L)	Ca (mg/dL)	PHO (mg/dL)	Globulin (g/dL)	A/G Ratio (g/dL)
Vehicle: analytical grade water with Tween 80 (about 0.5% w/v) and carboxymethyl cellulose (1% w/v)																						
G1*n* = 10	0		Mean	6.70	1.14	38.50	73.80	98.90	0.10	108.40	99.30	15.70	22.61	0.40	4.40	81.40	151.50	5.12	9.66	6.80	5.56	0.21
			±SD	0.33	0.12	10.22	9.90	13.92	0.00	26.73	13.47	2.98	4.30	0.07	1.07	32.85	4.43	0.29	0.25	0.67	0.29	0.02
Test article: micronized palmitoylethanolmide (particle size 0.5–10 *μ*m)																						
G2*n* = 10	250		Mean	7.29^a^	1.44	31.80	87.30	80.70^a^	0.10	95.80	113.80	15.80	22.75	0.44	5.20	48.60	144.80	4.59	10.19	5.86	5.85	0.25
			±SD	0.42	0.28	4.96	10.71	43.69	0.00	3.99	29.98	2.57	3.71	0.12	0.79	10.84	6.37	0.26	0.22	0.93	0.50	0.06
G3*n* = 10	500		Mean	6.98	1.44	27.80	83.80	57.00	0.10	100.90	110.30	15.30	22.03	0.33	4.50	58.50	142.50	4.48	10.16	6.21	5.54	0.26
			±SD	0.41	0.16	4.02	6.49	14.09	0.00	17.68	17.86	1.57	2.26	0.16	0.71	27.37	9.86	0.29	0.26	0.69	0.43	0.04
G4*n* = 10	1000		Mean	7.12^a^	1.48	31.60	86.10	52.70	0.10	97.60	119.00	15.40	22.18	0.41	4.30	65.50	148.30	4.66	10.04	6.04	5.64	0.26
			±SD	0.16	0.16	5.21	6.79	8.15	0.00	6.65	21.99	1.71	2.47	0.13	0.67	33.69	4.57	0.46	0.46	0.55	0.15	0.03

Male rats																									
End of recovery period (Day 119)																									
Group	Dose (mg/kg/day)			TP (g/dL)	ALB (g/dL)	ALT (IU/L)	AST (IU/L)		ALP (IU/L)		TBIL (mg/dL)		GLU (mg/dL)	CHOL (mg/dL)	BUN (mg/dL)	UREA (mg/dL)	CRE (mg/dL)	GGT (IU/L)	TG (mg/dL)	Na (mmol/L)	K (mmol/L)	Ca (mg/dL)	PHO (mg/dL)	Globulin (g/dL)	A/G Ratio (g/dL)
Vehicle control: analytical grade water with Tween 80 (about 0.5% w/v) and carboxymethyl cellulose (1% w/v)																									
G1 (R)*n* = 5	0	Mean		6.42	1.04	39.60		85.8015.39		73.20		0.20	91.00	93.80	14.40	20.74	0.30	4.60	109.00	142.60	5.00	9.22	6.66	5.38	0.19
		±SD		0.16	0.05	9.13				20.45		0.00	11.85	12.38	3.05	4.39	0.00	1.14	29.55	2.07	0.37	0.23	0.53	0.13	0.01
Test article: micronized palmitoylethanolamide (particle size 0.5–10 *μ*m)																									
G4 (R)*n* = 5	1000		Mean	6.36	1.04	58.80^a^		102.8		97.00		0.24	83.00	71.20^a^	15.80	22.75	0.28	5.40	108.20	143.00	5.20	9.02	6.38	5.32	0.20
			±SD	0.44	0.09	7.79		10.64		12.79		0.05	7.58	7.05	1.48	2.14	0.11	1.14	55.28	1.58	0.66	0.68	0.52	0.42	0.02

Female rats																									
End of recovery period (Day 119)																									
Group	Dose (mg/kg/day)		TP (g/dL)	ALB (g/dL)	ALT (IU/L)	AST (IU/L)		ALP (IU/L)		TBIL (mg/dL)	GLU (mg/dL)	CHOL (mg/dL)	BUN (mg/dL)	UREA (mg/dL)		CRE (mg/dL)		GGT (IU/L)	TG (mg/dL)	Na (mmol/L)	K (mmol/L)	Ca (mg/dL)	PHO (mg/dL)	Globulin (g/dL)	A/G Ratio (g/dL)
Vehicle control: analytical grade water with Tween 80 (about 0.5% w/v) and carboxymethyl cellulose (1% w/v)																									
G1 (R)*n* = 5	0	Mean	7.18	1.36	25.40	70.60		66.20		0.30	98.80	128.60	12.40	17.86		0.40		3.80	38.80	148.80	4.72	9.58	5.60	5.82	0.24
		±SD	0.46	0.29	4.83	6.23		18.53		0.00	9.50	40.32	2.61	3.76		0.07		0.84	6.14	6.06	0.33	0.30	0.48	0.54	0.06
Test article: micronized palmitoylethanolamide (particle size 0.5–10 *μ*m)																									
G4 (R)*n* = 5	1000	Mean	7.28	1.40	46.00^a^		103.40^a^		94.20	0.30	97.20	98.00	17.60^a^		25.34^a^		0.42	4.80	51.80	145.00	4.68	9.76^a^	5.42	5.88	0.24
		±SD	0.54	0.16	8.12		7.96		33.79	0.00	9.31	15.95	2.88		4.15		0.04	0.45	14.89	1.22	0.26	0.17	0.78	0.43	0.02

TP, total protein; ALB, albumin; ALT, alanine aminotransferase; AST aspartate aminotransferase; ALP, alkaline phosphatase, TBIL, total bilirubin, GLU, glucose; CHOL, total cholesterol; BUN, blood urea nitrogen; UREA, urea, calculated; CRE, creatinine; GGT, gamma glutamyltranspeptidase; TG, triglycerides; Na, sodium; K, potassium; Ca, calcium; PHO, phosphorus; A/G ratio, globulin, albumin/globulin ratio (calculated).

a Significantly different from control group (*P* < 0.05).

There were no differences in organ or tissue weights attributable to 90 days of treatment with microPEA. The only statistically significant difference in mean organ weights noted at end of the 90‐day treatment was that of the relative, but not absolute, heart weight in females being lesser than the control, which in absence of dose dependence, was considered to be incidental. At the end of the 28‐day recovery period, absolute and relative adrenal weights only in male rats were statistically higher in G4(R) as compared to G1(R) controls (Tables 9 and 10), and absolute liver weights were significantly lower in G4(R) females only than in G1(R) control rats (Table 9). These results appear to be incidental due to the lack of any changes in other correlated parameters, such as necropsy findings, histopathology findings, clinical hematology, and clinical chemistry.

**Table 9:** Summary of absolute organ weights (end of recovery period) (g)

Male rats												
Group	Dose mg/kg/day		Fasting body Wt. (g)	Adrenals	Testes	Kidneys	Liver	Brain	Thymus	Heart	Spleen	Epididymides
G1 (R)*n* = 5	0	Mean	483.60	0.044	3.77	4.10	14.87	2.44	0.55	1.64	0.84	1.41
		±SD	34.09	0.006	0.40	0.58	1.89	0.20	0.08	0.32	0.14	0.18
G4 (R)*n* = 5	1000	Mean	434.60	0.057^a^	3.66	3.35	12.45	2.31	0.46	1.50	2.02	1.31
		±SD	19.27	0.006	0.30	0.50	1.91	0.17	0.14	0.24	1.23	0.16

Female rats												
Group	Dose mg/kg/day		Fasting body Wt.	Adrenals	Ovaries	Kidneys	Liver	Brain	Thymus	Heart	Spleen	Uterus
G1 (R)*n* = 5	0	Mean	288.00	0.076	0.154	2.19	8.46	2.27	0.36	1.22	0.87	1.33
		±SD	31.96	0.016	0.029	0.09	0.40	0.12	0.08	0.12	0.47	0.84
G4 (R)*n* = 5	1000	Mean	279.40	0.064	0.138	2.04	7.44^a^	2.10	0.29	1.09	0.93	1.08
		±SD	7.64	0.008	0.013	0.37	0.68	0.15	0.03	0.07	0.44	0.51

a Organ weights of treated rats differ significantly (*P* > 0.05) from those of the control group.

**Table 10:** Summary of relative organ weights at end of recovery period (%)

Male rats												
Group	Dose mg/kg/day		Fasting body Wt. (g)	Adrenals	Testes	Kidneys	Liver	Brain	Thymus	Heart	Spleen	Epididymides
G1 (R)*n* = 5	0	Mean	483.60	0.009	0.78	0.85	3.07	0.51	0.11	0.34	0.17	0.29
		±SD	34.09	0.001	0.04	0.10	0.29	0.03	0.02	0.06	0.02	0.03
G4 (R)*n* = 5	1000	Mean	434.60	0.013^a^	0.84	0.77	2.86	0.53	0.11	0.34	0.46	0.30
		±SD	19.27	0.001	0.07	0.12	0.37	0.05	0.03	0.05	0.28	0.04

Female rats												
Group	Dose mg/kg/day		Fasting body Wt.	Adrenals	Ovaries	Kidneys	Liver	Brain	Thymus	Heart	Spleen	Uterus
G1 (R)*n* = 5	0	Mean	288.00	0.026	0.054	0.77	2.97	0.80	0.12	0.43	0.30	0.45
		±SD	31.96	0.005	0.012	0.09	0.40	0.08	0.03	0.06	0.17	0.23
G4 (R)*n* = 5	1000	Mean	279.40	0.023	0.049	0.73	2.67	0.75	0.10	0.39	0.34	0.39
		±SD	7.64	0.003	0.006	0.15	0.27	0.07	0.01	0.03	0.17	0.19

a Organ weights of treated rats differ significantly (*P* > 0.05) from those of the control group.

Gross necropsy revealed an absence of any remarkable gross abnormalities in all but two male animals at the end of the 90‐day treatment period and two males at the end of the 28‐day recovery phase. One G3 rat had multiple abscesses in the lungs and another G3 animal had underweight testes and epididymides following 90 days of treatment. These incidental findings were found only in the mid‐dose group and are concluded to be unrelated to dosing with microPEA. Moderate splenic enlargement was found in two G4(R) males at the end of the recovery period, but subsequent histopathologic examination showed this to be due to splenic congestion, a common condition considered to be incidental in this case (Intox, 2016) in the absence of any other causal factors (Cesta et al., 2014).

Histopathological evaluation of tissues and organs listed above, from control (G1) and high‐dose (G4) groups, showed no incidence of any remarkable findings that could be related to treatment due to lack of any dose dependency as well as their values falling within ranges of the laboratory's historical controls. Single animals in different groups showed the following isolated (1 in 20 animals) findings in the kidney, known to be common background lesions: tubular dilatation (G1 female); focal tubular regeneration (G4 male) or degeneration (G4 female); and lymphocytic infiltration (G1 male). Two high‐dose females showed a low incidence of renal tubular hyaline casts which was not associated with any other pathology in the kidney or kidney weight, or were there any associated effects in clinical chemistry or hematology parameters, body weight, or food consumption. These findings are considered to be incidental and of no biological significance. Microscopic changes in the lungs, thought to be due to accidental aspiration of control or test formulations during gavage administration, and thus not treatment related, including: minimal‐to‐mild perivascular aggregation of lymphocytes in 8 of 20 control animals (G1) and in 6 of 20 high‐dose animals (G4); mild peribronchial lymphoid tissue hyperplasia (3 in G1 compared to 4 in G4); alveolar macrophages in two animals each in G1 and G4; and minimal suppurative pneumonia (2 G1 males and 1 G4 male). Intestinal findings included isolated instances of minimal submucosal lymphoid hyperplasia in the terminal ileum (1 G1 male), colon (2 G1 males and 1 G4 male), and rectum (1 G1 male and 2 G4 females). Ectopic thymus, a developmental abnormality occasionally found in rodents that is generally unrelated to treatment, was observed in one control female (G1) and in two treated females (G4). The only other histopathological findings were in single control animals. Overall the histopathological evaluation showed no treatment‐related effects of any biological significance.

## Discussion

The results of the new toxicological studies described in this study show that microPEA has a favorable safety profile for its use in health products intended for human and companion animal consumption, with a no effect level in repeated dose studies that is >1000 mg/kg bw. These results are in agreement with earlier studies done with PEA by the then Czechoslovakia Academy of Sciences that were not published but are described in the review by Masek (1980). The first was an acute oral LD50 study using 1, 2, and 4 g/kg bw in the mouse. No deaths occurred in this acute toxicity study (Masek 1980). The second study was a chronic toxicity study in the mouse in which 0, 100, or 500 mg PEA per kg bw was administered to 50 mice in each group for 6 months (Masek 1980). At interim and terminal sacrifice, histological examination of lymph nodes, spleen, liver, lungs, and kidneys revealed some incidence of various lesions, but no difference in occurrence between treated and control groups. In the third study, a 6‐month chronic toxicity study in the rat, groups of 10 animals were exposed to 0, 100, and 500 mg PEA per kg bw (Masek 1980). Histological examination of kidneys, lymph nodes, liver, lungs, and spleen showed no differences in the incidence of adverse effects between the treated groups and the control. The fourth study examined embryotoxicity in pregnant mice that were given PEA at the dose 50 mg/kg bw for 12 days. No teratogenic or embryotoxic effects were found (Masek 1980). Finally, a 2‐month study was attempted in the dog (Masek 1980), but it suffered from too many deficiencies (e.g., a total of only seven animals, two dogs per treatment group, three dogs in the control group, background disease) to be considered as adequate for evaluation and no conclusions can be drawn. More recently, in an unpublished genetic toxicology study using the Salmonella bacteria reverse mutation assay (i.e., Ames Test), the results showed that PEA has no mutagenic activity in any of the five standard tester strains, either in the absence or with the presence of a mammalian metabolic activation system (Biolabs, 1992a). In additional unpublished toxicology studies by the same laboratory, all conducted in accordance with Good Laboratory Practice (GLP), PEA was shown to have low acute oral toxicity with an LD50 >5000 mg/kg body weight (bw) in the rat (Biolabs, 1992b), and was not sensitizing in a standard study using the guinea pig (Biolabs, 1992c). These studies are interpreted to mean that PEA (1) is not expected to show any genotoxic or carcinogenic potential, (2) has low oral toxicity, and (3) has no allergic sensitization potential.

These results of the GLP genotoxicity and rodent toxicology studies (Intox, 2015a,b,c,d, 2016) are in agreement with the aforementioned previous unpublished studies (Masek 1980, Biolabs, 1992a,b,c).

1. The genetox studies show a complete absence of mutagenic potential in Salmonella bacteria, interpreted to mean that the test substance is not likely to be a genotoxic carcinogen (OECD, 1997). The conclusion that microPEA is unlikely to be a genotoxic carcinogen is strengthened by the negative results of the micronucleus test, performed using human lymphocytes (Intox, 2015b). This test battery of two in vitro assays (i.e., the Ames and micronucleus tests) is either recommended or required by regulatory agencies worldwide, to investigate the carcinogenic potential of new substances during the premarket review and approval process.
2. The results from rodent studies show that the previous and present acute oral toxicity studies are in agreement (Masek 1980; Biolabs, 1992b; Intox, 2015c), and repeat oral toxicity studies consistently show an absence of adverse effects at the highest doses tested (Masek 1980, Intox, 2015d, 2016).

Although it is not the objective of this study to describe any aspect of the efficacy endpoints or findings of clinical studies, many human studies have been reported on the possible uses of PEA in the treatment and prevention of many types of illness and disorder. Initial tolerance studies were performed and described in the summary by Masek (1980). In one study, for example, five hospitalized children were given 50 mg PEA per kg bw for 2 weeks, and no side effects were observed. In another, 15 adults were given 100 mg PEA per kg bw for 3 weeks, and 12 biochemical parameters were examined before and after exposure to PEA. No adverse effects were found. Subsequently, two large studies were used to provide documentation for the approval of PEA as a drug (i.e., IMPULSIN^®^). In one featuring two trials (Masek et al. 1974), 1300 subjects were involved, about half receiving 30 mg/kg bw of PEA, and the other half a placebo, for a period of 12 consecutive days. Another study, using the same dosing regimen, involved more than 1800 subjects divided into three separate trials (Kahlich et al. 1979). No adverse side effects were reported in these studies.

It is noted, however, that most human studies have been done to examine the efficacy of PEA rather than its safety. It is also noted that the studies were short term, in the order of 2 weeks of consumption. Dozens of such studies have been done over a period of 40 years, which provides some indication of the safety of PEA. A reasonable number of recent human studies have been conducted by Italian investigators in the last few years, using a daily intake of 600 to 1200 mg, usually in two 300 mg installments. The test material in these studies mostly was identified as microPEA by the tradenames Normast^®^, Visimast^®^, or Pelvilen^®^ that are commercially available from the company Epitech in Saccolongo, Italy. Epitech is also the supplier of the microPEA used for the toxicity testing in this study. A study by Canteri et al. (2010) used three groups of subjects, including a placebo group, over a period of 21 days. This was a randomized double‐blind study with over 100 subjects: 35 in the placebo group, 38 receiving 300 mg/day, and 38 administered 600 mg of microPEA (Normast^®^ 300) per day. No adverse effects were observed. In a similar randomized, double‐blind study using Normast^®^ 300 and Normast^®^ 600 for 21 days, 209 volunteers (placebo group), 212 individuals (300 mg microPEA per day), and 215 others (600 mg microPEA/day), showed no adverse effects (Guida et al. 2010). In an open study without controls, Schifilliti et al. (2014) reported no adverse effects after 60 days in 30 subjects who were given 300 mg microPEA (Normast^®^ 300) twice a day. Bacci et al. (2011) conducted a 15‐day study with subjects acting as their own control. One adverse event was reported and considered to be irrelevant to microPEA (Normast^®^ 300) exposure of 300 mg, twice a day; 26 subjects completed the study. Pescosolido et al. (2011) administered 600 mg of microPEA (Visimast^®^) per day for 15 days. Subjects acted as their own controls, and no comment was made about possible side effects. Over a period of 2 months, 20 patients (10 males and 10 females) were given 300 mg of PEA (form and source not provided) twice a day in an observational study that did not report whether or not adverse events occurred (Truini et al. 2011). In a 6‐month study in 16 patients and in 16 nonblinded controls, side effects were reported not to have occurred following consumption of 300 mg of microPEA (Visimast^®^) twice a day (Costagliola et al. 2014). Additional studies have been published that use amounts in excess of 600 mg/day, including Marini et al. (2012) in which 12 subjects received 300 mg of microPEA (Epitech) twice a day for 7 days, and then 300 and 600 mg (total 900 mg) for 7 days. There was no placebo control, but another group of 12 subjects received ibuprofen, and it was reported that there were no side effects. Indraccolo and Barbieri (2010) administered 400 mg microPEA (Pelvilen^®^) twice a day (total 800 mg) to four subjects for 90 days. There were no controls, and the authors considered it “anecdotal” with a “total lack of side effects”. Calabro et al. (2010) reported a single case study, with no further details, in which the subject was given 1200 mg of PEA (form and source not reported) for 2 weeks. In a 30‐day trial, 12 (0 mg, but not placebo), 6 (600 mg microPEA), and 8 (1200 mg microPEA) subjects were reported to have no adverse effects after treatment with Normast^®^ 300 (Conigliaro et al. 2011). Assini et al. (2010), in a published note with no details or comments about safety, treated 25 subjects and 25 controls, with 600 mg PEA (form and source not provided) twice a day or 0 treatment (not placebo) for 60 days. Desio (2010) conducted an observational study with no control using 600 mg PEA (form and source not reported) twice a day for 45 days in 16 males and 14 females. There were no side effects.

As summarized above, a number of studies have been conducted with varying numbers of subjects and varying durations of daily microPEA consumption. Reviews of these (Skaper et al. 2014; Paladini et al. 2016) provide useful summaries of the efficacy of micronized PEA, as well as favorable comments on its safety. A common observation from the totality of the evidence described is the lack of adverse effects of doses as high as 1200 mg of microPEA per day. The most common regimen was 300 mg twice a day, although a sizeable body of evidence is accumulating on amounts 1200 mg/day. Adverse effects have been reported to be absent (Gatti et al. 2012; Skaper et al. 2014; Paladini et al. 2016).

In summary, available data from rodent and human studies support the safety of PEA in general, and of microPEA specifically, in products intended for human and companion animal consumption.

## Conflict of Interest

None declared.
